# Impact of long-term nitrogen scavenger therapy on clinical outcome in individuals with urea cycle disorders

**DOI:** 10.1038/s41598-026-42150-6

**Published:** 2026-03-18

**Authors:** Roland Posset, Friederike Epp, Sven F. Garbade, Florian Gleich, Andrea L. Gropman, Sandesh C. S. Nagamani, Georg F. Hoffmann, Stefan Kölker, Matthias Zielonka

**Affiliations:** 1 Medical Faculty Heidelberg, Heidelberg University, Heidelberg, Germany; 2 Division of Pediatric Neurology and Metabolic Medicine, Department I, Center for Pediatric and Adolescent Medicine, University Hospital Heidelberg, Im Neuenheimer Feld 430, 69120 Heidelberg, Germany; 3 Center for Experimental Therapeutics, St. Jude Children’s Research Hospital, Memphis, TN USA; 4 Department of Molecular and Human Genetics, Baylor College of Medicine and Texas Children’s Hospital, Houston, TX USA

**Keywords:** Sodium benzoate, Sodium/glycerol phenylbutyrate, Urea cycle disorders, UCDC, E-IMD, Diseases, Medical research, Neurology, Neuroscience

## Abstract

Principles of long-term medical management in individuals with urea cycle disorders (UCDs) encompass (1) a low protein diet, (2) supplementation of arginine and/or citrulline along with essential amino acids, nutrients, vitamins and trace elements, and (3) use of nitrogen scavenging agents to reduce recurrent hyperammonemic events (HAEs). These principles aim at providing metabolic stability, elimimation of chronic complications, and achievement of normal development as well as growth. A retrospective comparative analysis was performed by studying 138 individuals with male ornithine transcarbamylase deficiency (mOTC-D), citrullinemia type 1 (CTLN1) and argininosuccinic aciduria (ASA) based on *in vitro* residual enzymatic activity for severity-adjustment. Results show that individuals with mOTC-D, CTLN1 and ASA are at risk of progressive linear growth impairment, recurrent annual HAEs and an unfavorable neurocognitive outcome despite being under long-term nitrogen scavenging pharmacotherapy. No overall superiority among existing nitrogen scavenging agents with regard to the individual’s metabolic stability, linear growth impairment and poor neurocognitive outcome was observed. Novel therapeutic strategies are urgently needed to ultimately improve health outcomes in individuals with UCDs in order to sufficiently meet guideline-specific goals.

**Supplementary Information:**

The online version contains supplementary material available at 10.1038/s41598-026-42150-6.

## Introduction

Urea cycle disorders (UCDs) are a group of rare inborn errors of metabolism with a broad phenotypic variability ranging from severe disease courses with life-threatening episodes of hyperammonemic encephalopathy mostly during the first few days of life to attenuated disease forms. Attenuated phenotypes present with disease onset after the neonatal period and with less specific symptoms such as cognitive impairment, vomiting, behavioral or psychiatric abnormalities in the presence or absence of mild to moderate hyperammonemic events (HAEs)^1–4^. Prevention of HAEs by achieving stable metabolic control in individuals with UCDs is an important aim of long-term medical management, consisting of three main pillars, (1) low protein diet, (2) supplementation of arginine and/or citrulline along with essential amino acids, nutrients, vitamins and trace elements, and (3) pharmacotherapy to increase waste nitrogen excretion. Beyond that, a recent UCD guideline suggests that long-term medical management should optimally avoid chronic complications and maintain a normal development as well as growth^5^. However, little evidence is available to substantiate whether real-world long-term medical management indeed meets the above-mentioned guideline goals.

A recent analysis investigated the effects of medical management on growth in symptomatic individuals with UCDs and suggested that disease severity but not the degree of (iatrogenic) protein restriction is associated with postnatal linear growth impairment^6^, however this analysis was based on a clinical classification system for severity-adjustment and it lacked the proof of whether guideline goals are actually met by current medical management principles^5^. Importantly, a new strategy for severity-adjustment based on *in vitro* residual enzymatic activity for the assessment of intrinsic disease severity became available during the past years offering a translational research approach for explorative retrospective data analysis in individuals with male ornithine transcarbamylase deficiency, citrullinemia type 1 (CTLN1) and argininosuccinic aciduria (ASA)^7–9^. We used this research strategy to analyze whether real-world long-term pharmacotherapy with sodium benzonate (BZA) or sodium-/glycerol phenylbutyrate (PBA) alone or BZA and PBA in combination is indeed associated with sufficient achievement of guideline-specific goals (i.e. stable metabolic control, elimimation of chronic complications, and achievement of normal development as well as growth) in individuals with UCDs (i.e. mOTC-D, CTLN1, ASA) in a comparative manner.

## Results

### Description of the overall study sample

Overall, 138 individuals (mOTC: n = 48; CTLN1: n = 58; ASA: n = 32) were included in the analysis, out of which 70 individuals presented with an attenuated phenotype (mOTC-D: n = 48; CTLN1: n = 22) and 68 individuals presented with a severe phenotype (CTLN1: n = 36; ASA: n = 32). Age at first symptoms, age at diagnosis and initial peak plasma ammonium concentration (initial NH_4_^+^_max_) differed between the attenuated and severe phenotypes (*p* < 0.01 for age at first symptoms; *p* < 0.01 for age at diagnosis; *p* < 0.001 for initial NH_4_^+^_max_; each Two-sample Fisher-Pitman Permutation Test). Disease-specific analysis for CTLN1 revealed higher initial NH_4_^+^_max_ for the severe compared to the attenuated phenotype (*p* < 0.001; Two-sample Fisher-Pitman Permutation Test), whereas age at first symptoms and age at diagnosis did not differ between both phenotypes (*p* = 0.84 for age at first symptoms, *p* = 0.72 for age at diagnosis; Two-sample Fisher-Pitman Permutation Test; Suppl. Table 1).

Next, we studied whether use of a specific long-term scavenger treatment (i.e. BZA, or PBA, or BZA & PBA) was associated with age at first symptoms or age at diagnosis in attenuated or severe phenotypes and found no such association neither in the overall [*p* = 0.25 for age at first symptoms (attenuated phenotype); *p* = 0.64 for age at first symptoms (severe phenotype); *p* = 0.59 for age at diagnosis (attenuated phenotype); *p* = 0.63 for age at diagnosis (severe phenotype); each Two-sample Fisher-Pitman Permutation Test] nor in the disease-specific samples (Suppl. Table 2A and B). However, disease-specific analysis revealed an association between use of long-term bi-scavenger treatment (with BZA & PBA) and high initial NH_4_^+^_max_ in severely affected individuals with CTLN1 (*p* < 0.01; Two-sample Fisher-Pitman Permutation Test), reflecting that decision for a bi-scavenger therapy is associated with high initial peak plasma ammonium concentration rather than age at first symptoms or age at diagnosis in this group (Suppl. Table 2A–C).

### Endpoint analysis—HAEs per year of observation

**Fig. 1:**
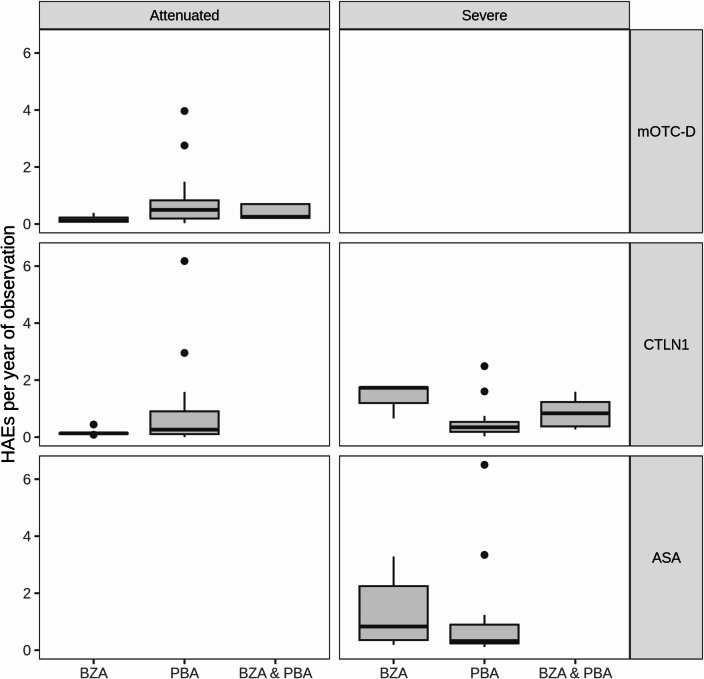
Number of HAEs per year of observation appears not to be associated with specific nitrogen scavenger treatment. Overall, use of a specific scavenger was not associated with reduced number of HAEs per year of observation (*p* = 0.18 for attenuated phenotype, *p* = 0.23 for severe phenotype; Two-sample Fisher-Pitman Permutation Test). However, for severely affected individuals with CTLN1, a trend towards reduced annual frequencies of HAEs was observed when PBA (as monotherapy or as part of a bi-scavenger therapy) was used for long-term management (*p* = 0.077; Two-sample Fisher-Pitman Permutation Test). Data are shown as median (black thick line) and mean (triangle), length of the box corresponds to the IQR, upper and lower whiskers correspond to max. 1.5 × IQR, each point represents an outlier. Boxplots were depicted, if three or more individuals received a specific therapy. ASA, argininosuccinic aciduria; BZA, sodium benzoate; CTLN1, citrullinemia type 1; mOTC-D, male ornithine transcarbamylase deficiency; PBA, sodium/glycerol phenylbutyrate. For descriptive characteristics see Supplementary Table 3.

We studied whether treatment with a specific scavenger or a bi-scavenger therapy might be associated with improved metabolic stability. Individuals with a severe phenotype of CTLN1 under long-term medical management with BZA suffer in mean from 1.38 HAEs per year of observation (n = 3), while their counterparts treated longitudinally with PBA (n = 18) or both BZA & PBA (n = 6) are confronted with 0.52 or 0.85 HAEs per year of observation in mean. Thus, long-term use of PBA (as monotherapy or as part of a bi-scavenger therapy) might be associated with a trend towards reduced HAEs per year of observation (*p* = 0.077; Two-sample Fisher-Pitman Permutation Test), suggesting that PBA might be beneficial with regard to metabolic long-term stability in severely affected individuals with CTLN1. No such association was found between long-term use of a specific scavenger (monotherapy or bi-scavenger treatment) and number of HAEs per year of observation for individuals with an attenuated phenotype of mOTC-D (*p* = 0.33; Two-sample Fisher-Pitman Permutation Test), an attenuated phenotype of CTLN1 (*p* = 0.31; Two-sample Fisher-Pitman Permutation Test), or individuals with a severe phenotype of ASA (each *p* = 0.53; Two-sample Fisher-Pitman Permutation Test) (Fig. 1, Suppl. Table 3).

### Endpoint analysis—motor abnormality

**Fig. 2:**
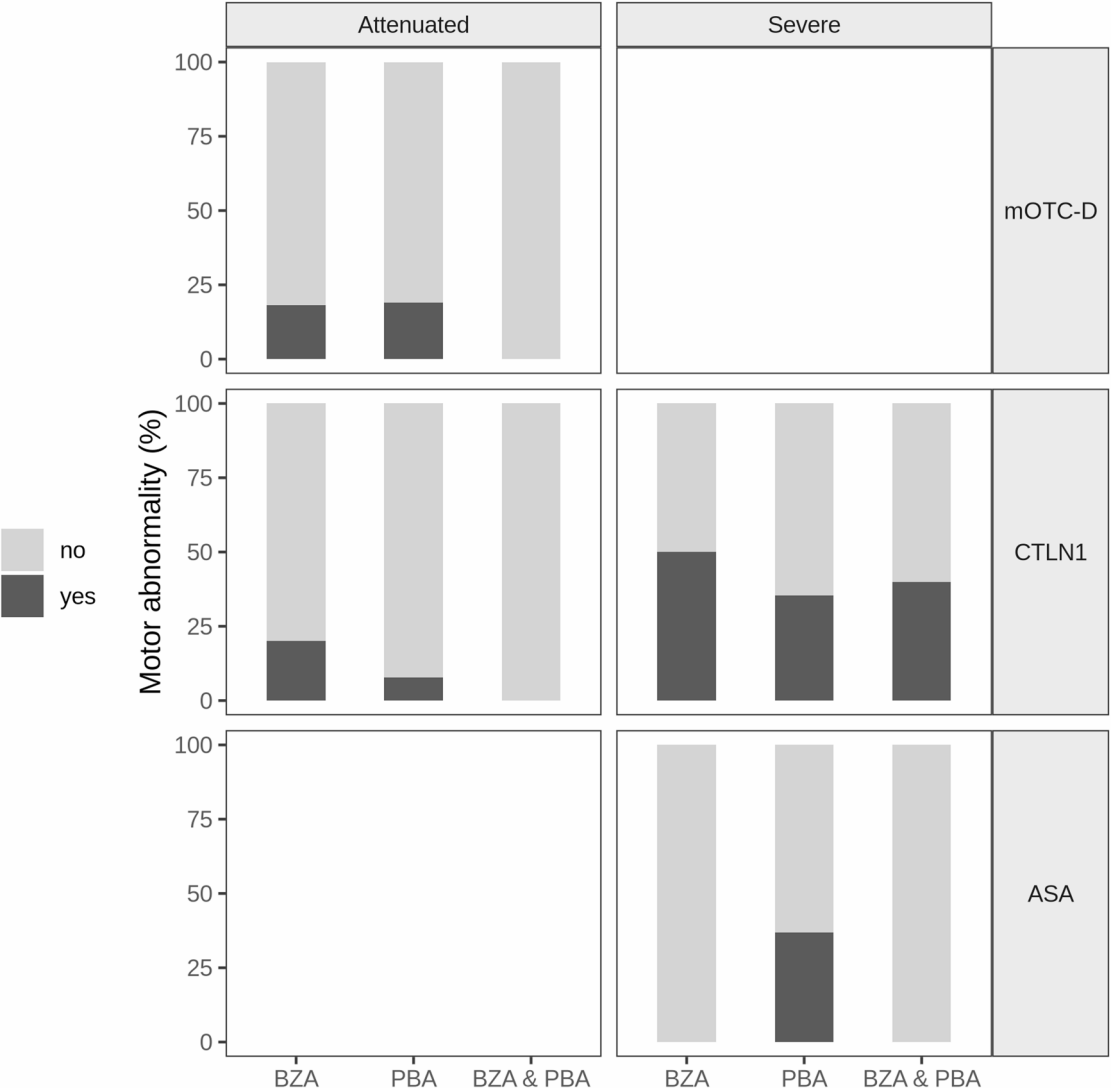
Motor abnormality appears to be independent from the long-term scavenger treatment. Boxplot illustrating the presence and absence of motor abnormality for individuals with mOTC-D, CTLN1 and ASA and an attenuated or a severe phenotype. Bright shading corresponds to the absence, dark shading corresponds to the presence of motor abnormalities at last follow-up within the IOP. No specific long-term scavenger treatment was superior over one or the other with regard to the motoric outcome (*p* = 0.44 for attenuated phenotype, *p* = 0.75 for severe phenotype; Pearson’s Chi-squared Test). Boxplots were depicted, if three or more individuals received a specific therapy. ASA, argininosuccinic aciduria; BZA, sodium benzoate; CTLN1, citrullinemia type 1; IOP, individual observation period; mOTC-D, male ornithine transcarbamylase deficiency; PBA, sodium/glycerol phenylbutyrate. For descriptive characteristics see Supplementary Table 4.

Hereafter, we investigated whether a specific long-term monoscavenger therapy (with BZA or PBA) or a bi-scavenger therapy (BZA & PBA) might be beneficial with regard to the neurological outcome as reflected by the presence or absence of motor abnormality at last regular follow-up visit within the individual observation periods (IOPs). Independent from the disease severity, no specific scavenger therapy (PBA, BZA or bi-scavenger therapy) was superior over one or the other with regard to the neurological outcome (*p* = 0.44 for attenuated phenotype, *p* = 0.75 for severe phenotype; Pearson’s Chi-squared Test), suggesting that the development of motor abnormalities in individuals with UCDs appears not to be associated with a specific long-term scavenger therapy (Fig. 2; Suppl. Table 4).

### Endpoint analysis—cognitive outcome

**Fig. 3:**
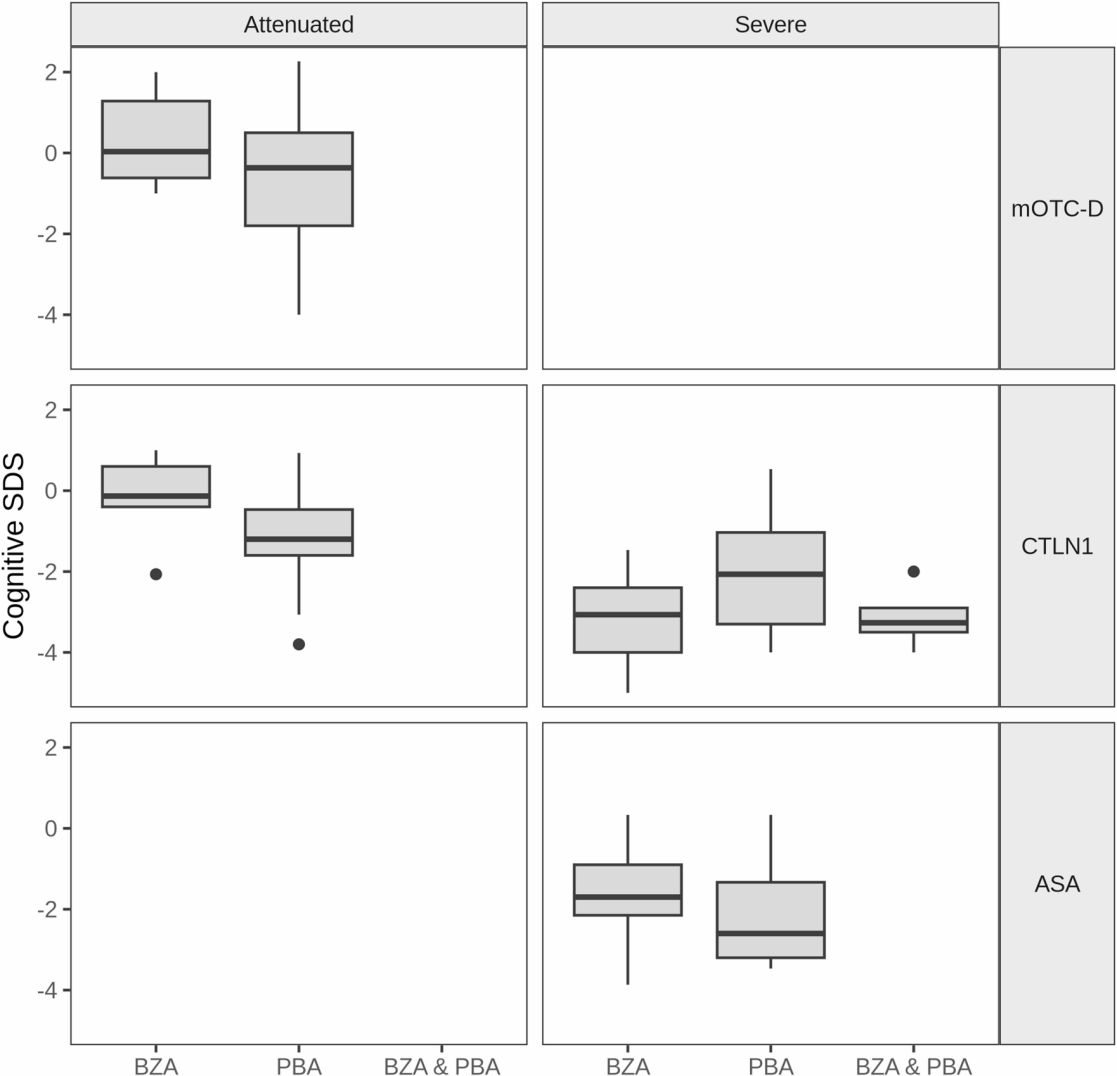
Cognitive outcome is not associated with aspecific long-term scavenger treatment. Disease-specific analysis (mOTC-D, CTLN1, ASA) showed that use of a specific scavenger as part of the long-term management is not associated with altered cognitive outcome at last follow-up within the IOP neither for individuals with an attenuated nor for individuals with a severe phenotype (*p* = 0.12 for attenuated mOTC-D, *p* = 0.26 for attenuated CTLN1, *p* = 0.23 for severe CTLN1, *p* = 0.35 for severe ASA; Two-sample Fisher-Pitman Permutation Test). Comparative analyses were performed in an age-adjusted manner. Data are shown as median (black thick line) and mean (triangle), length of the box corresponds to the IQR, upper and lower whiskers correspond to max. 1.5 × IQR, each point represents an outlier. Boxplots were depicted, if three or more individuals received a specific therapy. ASA, argininosuccinic aciduria; BZA, sodium benzoate; CTLN1, citrullinemia type 1; IOP, individual observation period; mOTC-D, male ornithine transcarbamylase deficiency; PBA, sodium/glycerol phenylbutyrate. For descriptive characteristics see Supplementary Table 5.

The cognitive outcome of age-adjusted individuals with an attenuated disease course (mOTC-D, CTLN1) was not associated with the use of a specific long-term monoscavenger treatment (BZA vs. PBA; *p* = 0.12 for mOTC-D, *p* = 0.26 for CTLN1; Two-sample Fisher-Pitman Permutation Test). Analogously, the cognitive outcome of age-adjusted individuals with a severe disease course (CTLN1, ASA) did not differ between the varying therapeutic subgroups applying either monoscavenger or bi-scavenger treatment (*p* = 0.23 for CTLN1, *p* = 0.35 for ASA; Two-sample Fisher-Pitman Permutation Test; Fig. 3, Suppl. Table 5), suggesting that disease severity rather than treatment with a specific nitrogen scavenger alone or in combination determines the cognitive outcome in individuals with mOTC-D, CTLN1 and ASA.

### Endpoint analysis—height development

Since a recent analysis suggested that progressive linear growth impairment—but not weight development—is associated with high intrinsic disease severity and with low concentrations of plasma branched chain amino acids (BCAAs) rather than with the degree of (iatrogenic) natural protein restriction^6^, we assessed whether a specific long-term scavenger treatment might be associated with a more favorable height development.

**Fig. 4:**
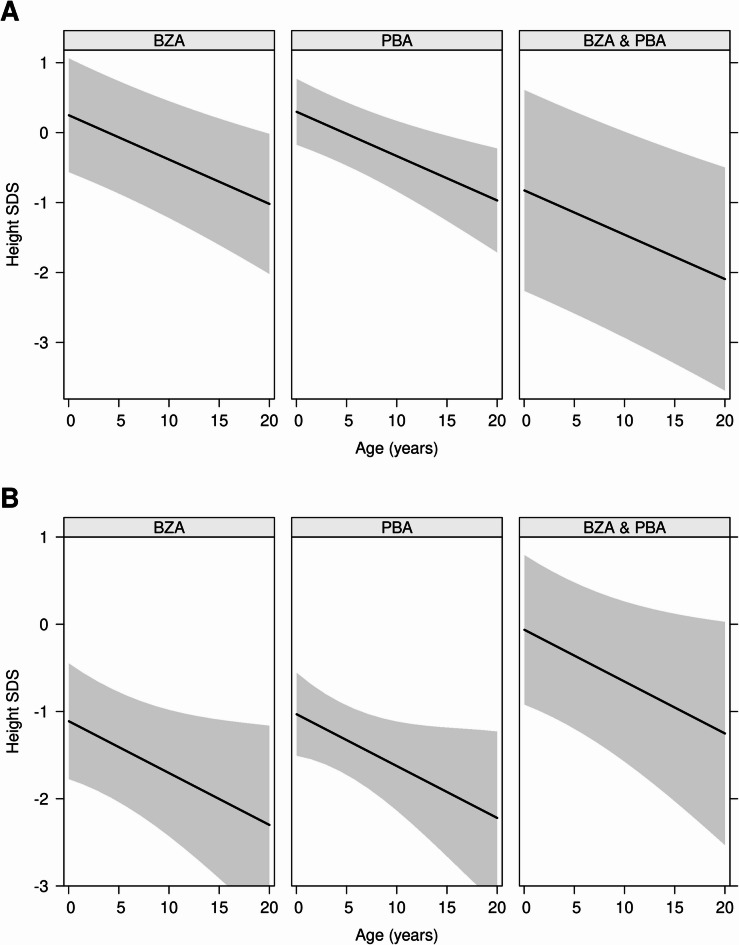
Postnatal linear growth impairment is not associated with the use of a specific scavenger treatment. Both individuals with an attenuated (**A**) and individuals with a severe phenotype (**B**) suffer from postnatal linear growth impairment within the IOPs. Importantly, age at follow-up (*p* < 0.001 for attenuated phenotype; *p* = 0.047 for severe phenotype; ANOVA), but not the underlying UCD subtype (*p* = 0.27 for attenuated phenotype; *p* = 0.34 for severe phenotype; ANOVA) or a specific long-term scavenger treatment (*p* = 0.34 for attenuated phenotype; *p* = 0.108 for severe phenotype; ANOVA) is associated with progressive linear growth impairment in both phenotypes. Since the underlying UCD subtype is not associated with linear growth impairment, individuals with mOTC-D, CTLN1 and ASA were pooled within each phenotypic category. Moreover, we could not identify different growth patterns during the observation periods, neither for the attenuated nor for the severe phenotype. Gray lines are fitted and height values from the LME model; the gray shaded area corresponds to 95% confidence interval. BZA, sodium benzoate; IOP, individual observation period; PBA, sodium/glycerol phenylbutyrate; SDS, standard deviation score. For descriptive characteristics see Supplementary Table 6.

We evaluated 97 severity-adjusted individuals [n = 42 with an attenuated disease course and n = 55 with a severe disease course] with regard to their height development. Interestingly, the age at follow-up (*p* < 0.001 for attenuated phenotype; *p* = 0.047 for severe phenotype; ANOVA), but not the underlying UCD subtype (*p* = 0.27 for attenuated phenotype; *p* = 0.34 for severe phenotype; ANOVA) or a specific long-term scavenger treatment (*p* = 0.34 for attenuated phenotype; *p* = 0.108 for severe phenotype; ANOVA) is associated with progressive growth impairment both in attenuated and severe phenotypes, supporting that (intrinsic) disease severity but not a specific disease or a long-term scavenger treatment appears to be associated with progressive linear growth impairment in individuals with UCDs (Fig. 4, Suppl. Table 6).

## Discussion

Using a recently established genotype-specific classification system for early prediction of disease severity in UCDs that has been proven to be beneficial in several comparative outcome analyses^10–13^, we systematically assessed the impact of long-term scavenger treatment on guideline-specific goals^5^. The present study has three main findings: (1) Annual frequency of HAEs is not associated with the use of a specific nitrogen scavenger. However, a potential trend towards reduced number of HAEs per year of observation might be observed in the severe phenotype of CTLN1, when PBA was part of long-term medical therapy either alone or in combination with BZA. (2) Long-term neurocognitive outcomes are independent from the use of a specific scavenging therapy, but rather reflect intrinsic disease severity. (3) All individuals are at risk of progressive linear growth impairment, that is independent from the use of a specific nitrogen scavenger.

Long-term pharmacotherapy with either PBA or BZA is essential to lower plasma NH_4_^+^ concentrations in individuals with UCDs aiming at preventing subsequent (brain-damaging) HAEs during the individual disease course^14,15^. Theoretically, PBA should be twice as efficient as BZA in deposing nitrogen on a mole-per-mole basis by complexing glutamine to form phenylacetylglutamine as opposed to the formation of hippuric acid by binding of glycine in the case of BZA^5^. Despite the biochemical superiority of PBA with regard to nitrogen excretion and ureagenesis, that has been demonstrated in healthy volunteers^16^, annual frequency of HAEs did not differ between the PBA- or BZA-treated groups of individuals with attenuated phenotypes of mOTC-D and CTLN1 as well as individuals affected by a severe disease form of ASA, suggesting equal efficacy of PBA and BZA on long-term metabolic stability in the respective UCD subtypes. Analogously, unchanged frequency of subsequent metabolic decompensations in UCDs were recently reported in individuals identified by newborn screening as well as dialyzed- or non-dialyzed cohorts after initial disease manifestation^12,13^.

However, PBA might be associated with a potential trend towards reduced number of HAEs in the severe phenotypic category of CTLN1 as opposed to BZA in the present study. Further prospective severity-adjusted analyses are needed to substantiate these findings given the retrospective nature and the limited number of included probands in this subanalysis.

Importantly, NH_4_^+^_max_ is a reliably predictor of neurocognitive outcome in UCDs^17,18^ and recurrent HAEs are thought to exert further detrimental effects on higher brain functions by disruption of the cerebral glutamate/glutamine cycle, neuronal bioenergetic impairment as well as hyperglutamatergic excitotoxicity^19–25^. Thus, improved neurocognitive outcome by the prevention of recurrent HAEs by efficient scavenging therapies could potentially be expected. However, none of the investigated scavenging agents (i.e. PBA, BZA or bi-scavenging therapy) was superior over one or the other with regard to the neurocognitive outcome as reflected by the unchanged frequency of motor abnormality and the unaltered cognitive SDS in severity- and age-adjusted analyses in the present study. Notably, cognitive SDS was determined by the intrinsic disease severity with severe phenotypes presenting with lower cognitive SDS at last follow-up visit when compared to their attenuated counterparts. These results are also corroborated in previous studies showing no differences in cognitive outcome in dialyzed and non-dialyzed cohorts who are subsequently treated by standard scavenging medical management^13,26,27^. These consistent observations might potentially be explained by the cognitive impairment being (mainly) caused by the devastating impact of hyperammonemic encephalopathy at initial disease manifestation and therefore might be considered a neurological long-term sequela of initial NH_4_^+^_max_, which typically is the most severe hyperammonemic decompensation in an individual’s lifespan, generally prior to the initiation of scavenger therapy. Thus, it might be questionnable, whether superiority of a specific scavenger therapy on neurocognitive outcome can be expected at all. Whether subsequent HAEs might relevantly contribute to cognitive impairment is subject to controversial debate and needs to be addressed in future research.

The limited therapeutic efficacy of current pharmacotherapy on neurocognitive outcome highlights an urgent need for new therapeutic approaches to mitigate the neuronal downstream cascade of neurotoxicity induced by elevated NH_4_^+^ concentrations. In light of the neurodegenerative disease course in CTLN1 and ASA that is thought to be attributed to ammonia-independent pathomechanisms due to the context-specific neuronal dysfunction of the argininosuccinate synthetase 1 and argininosuccinate lyase proteins^18,28–33^, disease-specific treatment strategies—beyond hyperammonemia—are indispensable to improve neurocognitive outcome in cytosolic UCDs as soon as the exact molecular events are unraveled.

Linear growth impairment in UCDs has recently been shown to be independent from the degree of therapeutic protein restriction, but to be associated with the intrinsic disease severity due to the consumption of BCAAs in acute and chronic hyperammonemia^6^. These findings could be corroborated in the present study with the identification of age-dependent progressive growth impairment in both the attenuated and severe phenotypic category, that was independent from the exact scavenging agent. Despite the fact, that PBA is known to be associated with decreased BCAA concentrations in blood^34^, treatment with PBA was not associated with more pronounced growth impairment when compared to the long-term treatment with BZA alone or in combination.

The present analysis has some inherent limitations. First, missing information on availability of medical care, concomitant treatment modalities, varying treatment protocols, that could not be controlled for in this study, might potentially have had an impact on the assessed outcome parameters. Second, further covariates such as presence, duration and severity of coma during the initial hyperammonemic decompensation as well as information on seizure presence, duration and severity could not be included due to low data density and quality of entered data in the respective E-IMD and UCDC databases. Third, longitudinal studies taking advantage of cerebral MR-imaging will be indispensable to assess the effect of long-term scavenger therapy on further important outcome parameters, such as but not limited to morphological signs of brain damage or alterations of cerebral metabolites applying *in vivo* spectroscopy, in a prospective manner*.* Fourth, some of the present analysis rely on a limited number of individuals. Therefore, the results in this analysis need to be confirmed in larger cohorts in future, optimally in a prospective manner. Importantly, the severe phenotypic category of mOTC-D and the attenuated phenotype of ASA could not be systematically assessed in the present study due to low density of information on scavenging therapy and/or too short treatment intervals for the respective subgroups that is explained by early death, liver transplantation or switch of scavenging treatment (for mOTC-D) and exclusive treatment with L-arginine supplementation (for ASA). Therefore, it remains elusive whether the observed findings might also apply for those two UCD-subgroups. Moreover, further (ultra-rare) UCD subtypes could not yet be included in the present analysis and remain subject to future research given the lack of analogous disease prediction models for severity stratification. In perspective, further analyses might replace initial NH_4_^+^_max_ as surrogate parameter for residual enzymatic activity, when genetic testing is performed for all individuals with UCDs followed by quantification of *in vitro* residual enzymatic activity thereby increasing analysis robustness.

## Conclusion

Use of nitrogen scavenging agents is a main pillar of long-term medical management in order to provide metabolic stability. However, the present comparative analysis using *in vitro* residual enzymatic activity for severity-adjustment suggests that independent from the use of a specific nitrogen scavenger, metabolic stability and clinical outcome is not satisfactory for the three most prevalent UCD subtypes (i.e. mOTC-D, CTLN1, ASA). Therefore, novel therapeutic strategies (i.e. prevention or attenuation of the initial HAE, prevention of recurrent HAEs, addressing alternative ammonia-independent pathomechanisms) are urgently needed to sufficiently meet guideline-specific goals and ultimately improve health outcomes in UCDs.

## Materials and methods

### Eligibility criteria and study sample

The data model of the UCDC and E-IMD registries and the research strategy for combined and comparative data analysis have been previously described^35–38^. Only individuals with confirmed diagnosis of mOTC-D, CTLN1 and ASA, who presented with initial hyperammonemic decompensation, with information on initial NH_4_^+^_max_ available, were included in this analysis. Requirements set forth by the International Committee of Medical Journal Editors were met. All procedures were in accordance with the ethical standards of the Helsinki Declaration of 1975, as revised in 2013. Written informed consent was obtained from the probands or their legal representatives prior to enrollment to this study. The respective UCDC and E-IMD study sites received written approval from an Institutional Review Board or Research Ethics Committee. Data were retrieved from the UCDC and E-IMD electronic databases as previously reported^10,11^. Cut-off date for the data pull was April 16^th^, 2025.

### Severity-adjustment and stratification

Severity-adjustment was carried out as discussed earlier^11^. In brief, individuals with mOTC-D, CTLN1 and ASA were categorized according to previously described models to predicting individual disease severity based on the pathogenic variant(s) in *OTC*, *ASS1* or *ASL*^7–9^. To this end, individuals with residual enzymatic OTC, ASS1 or ASL activities as assessed by the recently described genotype-specific *in vitro* system below the threshold values of 4.3% (for mOTC-D), 8.1% (for CTLN1) and 7.9% (for ASA), respectively, were classified as “severe”. Individuals with residual enzymatic activities equal to or above these thresholds were labeled as “attenuated” disease form. Due to the strong correlation between initial NH_4_^+^_max_ and residual enzymatic activities^7–9^, post-hoc posterior simulation was used by applying the mathematical correlation functions of initial NH_4_^+^_max_ with residual enzymatic activities (i.e. OTC, ASS1, ASL) to determine estimated initial NH_4_^+^_max_ values corresponding to the respective enzymatic threshold values for phenotypic differentiation. For this intention, 200 predicted initial NH_4_^+^_max_ values from fitted generalized additive models (GAM) were drawn and the following initial NH_4_^+^_max_ values were identified (50^th^ centile): 564 µmol/L for OTC-D (corresponding to residual enzymatic OTC activity of 4.3%), 303 µmol/L for CTLN1 (corresponding to residual enzymatic ASS1 activity of 8.1%) and 131 µmol/L for ASA (corresponding to residual ASL activity of 7.9%; Supplementary Fig. 1). Then, initial NH_4_^+^_max_ as surrogate marker for residual enzymatic OTC, ASS1 or ASL activity was used for group assignment of individuals to their underlying phenotypic severity to increase the number of included and investigated individuals.

### Biochemical and clinical variables used for data analyses

Subsequent numerical variables were included in this data analysis: Initial NH_4_^+^_max_, age at first symptoms, and age at diagnosis. The following clinical endpoints were studied: Number of HAEs (NH_4_^+^_max_ > 100 µmol/L) per year of observation, motor abnormality as superordinate dichotomous variable (yes/no) including several motor variables (dystonia, chorea, ataxia, spasticity, abnormal gross or fine motor function, delayed milestones, muscular hypotonia or hypertonia) in analogy to previous publications^10,11,13,17^. Cognitive outcome was assessed using cognitive standard deviation scores (SDS) at the most recent study visit. Cognitive SDS was calculated by applying cognitive scale from the Wechsler Adult Intelligence Scale, Wechsler Intelligence Scale for Children, Wechsler Preschool and Primary Scale of Intelligence, Bayley Scales of Infant Development and Adaptive Behavior Assessment Scale.

With regard to height development, all investigated individuals were normalized to standard deviation scores from UK growth charts^39^. Z-scores for height were calculated at each regular follow-up visit during the IOPs. Individuals were not stratified according to their protein intake since a recent analysis showed no association between degree of (iatrogenic) protein restriction and growth^6^.

Each individual was assigned to a long-term scavenger treatment group (i.e. BZA-group, PBA-group, or BZA & PBA-group) and the clinical endpoints (motor abnormality, cognitive outcome) were studied at the end of the IOPs (last regular follow-up visit), whereas height SDS was studied at each regular follow-up visit within the IOPs. IOPs comprised a follow-up period of ≥ 1 year and were censored if (1) liver transplantation occurred, or (2) a permanent change (de-/escalation of pharmacotherapy) or switch in long-term scavenger treatment occurred. Past hyperammonemic events were included in this analysis, thus the number of HAEs per year of observation were studied throughout the study time (i.e. time between the date of birth and the last study visit).

### Comparative analysis of long-term scavenger treatment on validated health-related outcome parameters

By use of above-mentioned methodological approach, the impact of long-term monoscavenger (BZA; PBA) or bi-scavenger (BZA & PBA) treatment is systematically assessed on above mentioned health-related clinical outcome parameters and stratified according to the individual’s disease severities (attenuated vs. severe phenotype). Since severely affected individuals with mOTC-D to a larger extent either (a) died or received LTx before being included into the databases, or (b) switched scavenger treatment, died or were liver transplanted within the first year of follow-up (exclusion criteria), systematic analysis of mOTC-D individuals with a severe disease course was not feasible, due to small sample sizes. Additionally, since attenuated individuals with ASA did not regularly receive long-term scavenger treatment, those individuals are not represented in this study, likewise due to small sample sizes. Thus, this study focuses on individuals with mOTC-D and CTLN1 and an attenuated disease course as well as individuals with CTLN1 and ASA and a severe disease course.

### Data availability

The UCDC database is registered at the US National Library of Medicine (https://clinicaltrials.gov), whereas the E-IMD registry is recorded on the German Clinical Trials Register (https://www.drks.de). Due to existing data protection laws, the datasets generated and analyzed during the current study are not publicly available. Data ownership is retained by the members of the UCDC and E-IMD consortia study group. Data availability is subject to the consent of both consortia upon request.

### Statistical analysis

Statistical analysis was computed with R software (version 4.5.2, R Core Team, 2025). For descriptive statistics, mean, standard deviation, median, interquartile range, and range were calculated, until otherwise stated. Dependent variables (e.g., age at diagnosis, initial NH_4_^+^_max_) and endpoints (e.g., HAEs per year of observation, cognitive SDS) were tested separately for attenuated and severe disease courses. Motor abnormality (no/yes) was analyzed with a χ2-test. Non-paired numeric data between groups (e.g. between scavenger treatment) were tested with a Fisher-Pitman Permutation Test for two- or k-samples (R package ‘coin’, version 1.4–3). Longitudinal growth data were analyzed with a linear mixed effect model (LME, function ‘lme’ from R package ‘nlme’, version 3.1.168), and we report p-values from analysis of variance table (ANOVA) with marginal sum of squares (function ‘anova.lme’ from R package ‘nlme’). We modeled LMEs with random slope and random intercept for age in individuals. In LME, predictor variables were age in years at anthropometric measurement, scavenger treatment (BZA, PBA or BZA & PBA) and UCD subtype (mOTC-D, CTLN1, or ASA). Effects graphics for LMEs were created using R package ‘effects’ version 4.2–4.

## Supplementary Information

Supplementary Information.

## Abbreviations

ASA: Argininosuccinic aciduria
BCAAs: Branched chain amino acids
BZA: Sodium benzoate
cSDS: Cognitive standard deviation score
CTLN1: Citrullinemia type 1
E-IMD: European registry and network for intoxication type metabolic diseases
IOP(s): Individual observation period(s)
initial NH_4_^+^_max_: Initial peak plasma ammonium concentration
IQR: Interquartile range
mOTC-D: Male ornithine transcarbamylase deficiency
PBA: Sodium/glycerol phenylbutyrate
SD(S): Standard deviation (score)
UCDC: Urea cycle disorders consortium
UCD(s): Urea cycle disorder(s)
