# Supplementary material for: Impact of long-term nitrogen scavenger therapy on clinical outcome in individuals with urea cycle disorders
Source: Sci Rep. 2026 Mar 18;16:19531. doi: 10.1038/s41598-026-42150-6 (PMC13291364; doi:10.1038/s41598-026-42150-6)
Supplement: Supplementary file 1 — Supplementary Information. [file 41598_2026_42150_MOESM1_ESM.pdf]

Suppl. Figure 1

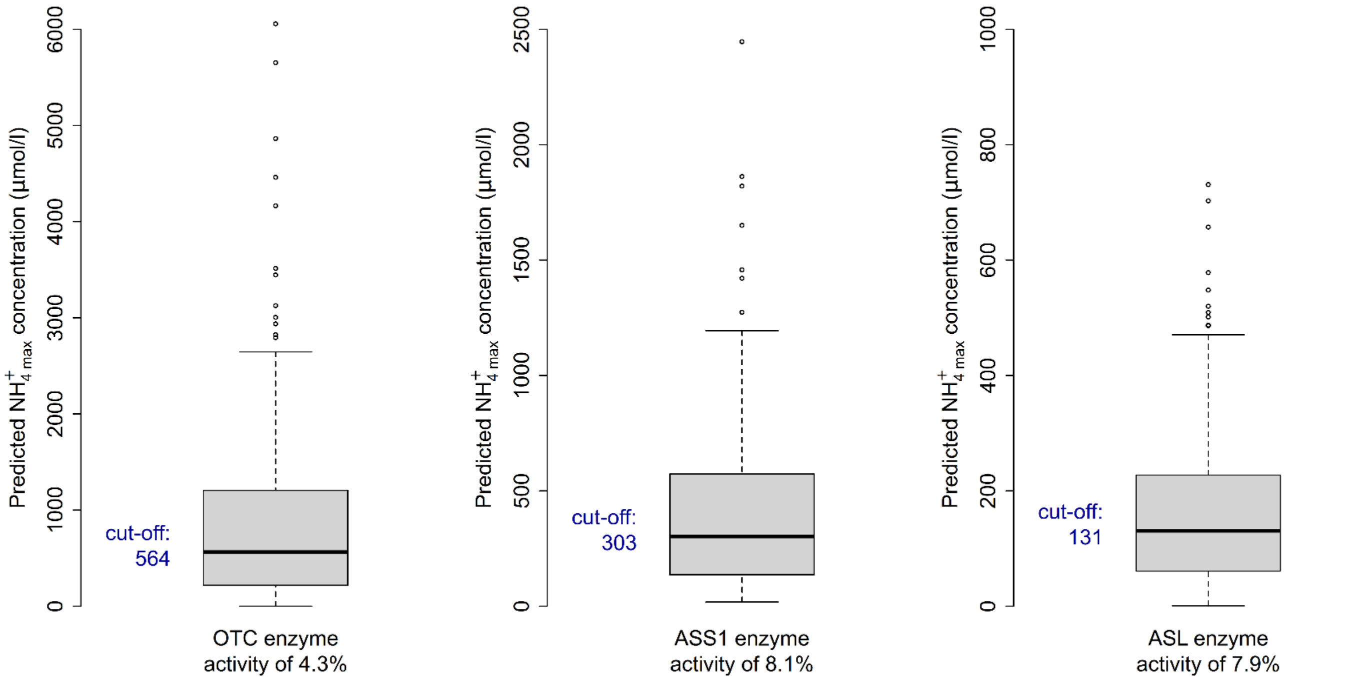

Suppl. Figure 1. Post-hoc statistical posterior simulation. Predicted initial $NH_4^+{}_{max}$ (50th centile; in µmol/L) correspond(s) to the disease-specific threshold value(s) of residual enzymatic activity/-ies differentiating individuals with an attenuated and severe phenotype with mOTC-D, CTLN1 and ASA. Corresponding to the residual enzymatic acitivity/-ies of ≤ 4.3 % (mOTC-D), or ≤ 8.1% (CTLN1), or ≤ 7.9% (ASA), initial $NH_4^+{}_{max}$ ≥ 564 µmol/L (mOTC-D), or ≥ 303 µmol/L (CTLN1), or ≥ 131 µmol/L (ASA) are defined as respective cut-off values and therefore as severe phenotypes. Data are shown as median (black thick line) corresponding to the cut-off value for the surrogate parameter initial $NH_4^+{}_{max}$, length of the box corresponds to interquartile range (IQR), upper and lower whiskers correspond to max. 1.5 x IQR, each point represents a simulated outlier. ASL, argininosuccinate lyase; ASS1, argininosuccinate synthetase 1, OTC, ornithine transcarbamylase. Data was adapted from [11].

Suppl. Table 1. Descriptive characteristics of the overall study sample

Overall study cohort		
	**Age at first symptoms [days]**<br>Mean, SD<br>Median, Min, Max, n	
	**Attenuated**	**Severe**
**Overall**	1245, 2933<br>287, 0, 21900, 70	95, 603<br>3, 1, 4957, 68
**mOTC-D**	1759, 3424<br>408, 0, 21900, 48	n.a.
**CTLN1**	122, 325<br>4, 1, 1460, 22	160, 827<br>3, 1, 4957, 36
**ASA**	n.a.	22, 58<br>4, 1, 275, 32
	**Age at diagnosis [days]**<br>Mean, SD<br>Median, Min, Max, n	
	**Attenuated**	**Severe**
**Overall**	1348, 3014<br>350, 0, 21900, 70	99, 605<br>5, 0, 4956, 68
**mOTC-D**	1922, 3500<br>480, 1, 21900, 48	n.a.
**CTLN1**	96, 178<br>6, 0, 610, 22	161, 829<br>4, 2, 4957, 36
**ASA**	n.a.	30, 82<br>5, 0, 360, 32
	**Initial $NH_4^+{}_{max}$ [µmol/L]**<br>Mean, SD<br>Median, Min, Max, n	
	**Attenuated**	**Severe**
**Overall**	242, 121<br>209, 52, 552, 70	834, 655<br>689, 136, 3600, 68
**mOTC-D**	269, 131<br>240, 82, 552, 48	n.a.
**CTLN1**	183, 69<br>183, 52, 301, 22	929, 530<br>714, 307, 2060, 36
**ASA**	n.a.	728, 767<br>489, 136, 3600, 32

Descriptive characteristics of the overall study sample. In the overall sample, age at first symptoms ($p<0.01$), age at diagnosis ($p<0.01$) and initial peak plasma ammonium concentration ($p<0.001$) differed between the attenuated and severe phenotypes. Disease-specific analysis for CTLN1 revealed higher initial peak plasma ammonium concentrations for severe compared to attenuated phenotypes ($p<0.001$), whereas age at first symptoms and age at diagnosis did not differ ($p=0.84$ for age at first symptoms, $p=0.72$ for age at diagnosis). Two-sample Fisher-Pitman Permutation Test was applied. ASA, argininosuccinic aciduria; CTLN1, citrullinemia type 1; initial $NH_4^+{}_{max}$, initial peak plasma ammonium concentration; mOTC-D, male ornithine transcarbamylase-deficiency; SD, standard deviation.

Suppl. Table 2A. Scavenger treatment in the overall study sample – age at first symptoms

Age at first symptoms [days]<br>Mean, SD<br>Median, Min, Max, n			
**Attenuated**			
	**Benzoate**	**Butyrate**	**Benzoate & Butyrate**
**Overall**	971, 1417<br>305, 2, 4745, 17	1683, 3664<br>365, 1, 21900, 41	134, 243<br>4, 0, 730, 12
**mOTC-D**	1341, 1550<br>480, 6, 4745, 12	2472, 4322<br>730, 2, 21900, 27	178, 269<br>7, 0, 730, 9
**CTLN1**	82, 131<br>7, 2, 305, 5	162, 399<br>5, 1, 1460, 14	2, 1<br>3, 1, 3, 3
**ASA**	n.a.	n.a.	n.a.
**Severe**			
	**Benzoate**	**Butyrate**	**Benzoate & Butyrate**
**Overall**	13, 43<br>2, 1, 4957, 17	151, 774<br>4, 1, 4957, 41	3, 1<br>3, 1, 5, 10
**mOTC-D**	n.a.	n.a.	n.a.
**CTLN1**	3, 2<br>2, 1, 6, 8	285, 1105<br>3, 1, 4957, 20	3, 1<br>3, 1, 4, 8
**ASA**	22, 59<br>3, 1, 180, 9	23, 62<br>4, 2, 275, 21	4, 1<br>4, 3, 5, 2

Descriptive characteristics (age at first symptoms) of the overall study sample stratified by scavenger treatment. In the overall sample, age at first symptoms was not associated with use of a specific scavenger treatment neither for the attenuated nor for the severe phenotypes ($p=0.25$ for attenuated phenotype; $p=0.64$ for severe phenotype). Likewise, disease-specific analyses revealed no association between age at first symptoms and use of a specific scavenger treatment neither for the attenuated nor for the severe phenotypes (each $p>0.05$ for attenuated mOTC-D, for attenuated CTLN1, for severe CTLN1, and for severe ASA). Two-sample Fisher-Pitman Permutation Test was applied. ASA, argininosuccinic aciduria; CTLN1, citrullinemia type 1; mOTC-D, male ornithine transcarbamylase-deficiency; SD, standard deviation.

Suppl. Table 2B. Scavenger treatment in the overall study sample – age at diagnosis

**Age at diagnosis [days]**<br>Mean, SD<br>Median, Min, Max, n			
**Attenuated**			
	**Benzoate**	**Butyrate**	**Benzoate & Butyrate**
**Overall**	1105, 1613<br>420, 2, 4745, 17	1640, 3664<br>397, 0, 21900, 41	695, 1865<br>41, 1, 6570, 12
**mOTC-D**	1518, 1773<br>480, 9, 4745, 12	2437, 4326<br>730, 3, 21900, 27	919, 2134<br>126, 1, 6570, 9
**CTLN1**	114, 148<br>14, 2, 305, 5	105, 207<br>5, 0, 610, 14	24, 31<br>9, 3, 60, 3
**ASA**	n.a.	n.a.	n.a.
**Severe**			
	**Benzoate**	**Butyrate**	**Benzoate & Butyrate**
**Overall**	17, 43<br>5, 2, 180, 17	156, 778<br>5, 0, 4957, 41	4, 2<br>4, 3, 8, 10
**mOTC-D**	n.a.	n.a.	n.a.
**CTLN1**	8, 9<br>6, 2, 30, 8	284, 1109<br>4, 2, 4957, 20	4, 2<br>4, 3, 8, 8
**ASA**	24, 58<br>5, 3, 180, 9	35, 95<br>5, 0, 360, 21	4, 1<br>4, 3, 5, 2

Descriptive characteristics (age at diagnosis) of the overall study sample stratified by scavenger treatment. In the overall sample, age at diagnosis was not associated with use of a specific scavenger treatment neither for the attenuated nor for the severe phenotypes ($p=0.59$ for attenuated phenotype; $p=0.63$ for severe phenotype). Likewise, disease-specific analyses revealed no association between age at diagnosis and use of a specific scavenger treatment neither for the attenuated nor for the severe phenotypes (each $p>0.05$ for attenuated mOTC-D, for attenuated CTLN1, for severe CTLN1, and for severe ASA). Two-sample Fisher-Pitman Permutation Test was applied. ASA, argininosuccinic aciduria; CTLN1, citrullinemia type 1; mOTC-D, male ornithine transcarbamylase-deficiency; SD, standard deviation.

Suppl. Table 2C. Scavenger treatment in the overall study sample – Initial $NH_4^+{}_{max}$

Initial $NH_4^+{}_{max}$ [µmol/L]<br>Mean, SD<br>Median, Min, Max, n			
**Attenuated**			
	**Benzoate**	**Butyrate**	**Benzoate & Butyrate**
**Overall**	266, 144<br>242, 54, 500, 17	246, 115<br>224, 52, 552, 41	196, 105<br>158, 113, 431, 12
**mOTC-D**	304, 150<br>340, 82, 500, 12	279, 122<br>240, 110, 552, 27	194, 114<br>139, 113, 431, 9
**CTLN1**	175, 78<br>185, 54, 265, 5	182, 65<br>176, 52, 282, 14	204, 93<br>194, 116, 301, 3
**ASA**	n.a.	n.a.	n.a.
**Severe**			
	**Benzoate**	**Butyrate**	**Benzoate & Butyrate**
**Overall**	987, 864<br>700, 142, 3600, 17	662, 494<br>550, 136, 2279, 41	1284, 613<br>1246, 450, 2060, 10
**mOTC-D**	n.a.	n.a.	n.a.
**CTLN1**	810, 404<br>702, 387, 1585, 8	770, 437<br>655, 307, 1700, 20	1146, 576<br>1415, 450, 2060, 8
**ASA**	1144, 1136<br>700, 142, 3600, 9	558, 533<br>386, 136, 2279, 21	639, 157<br>639, 528, 750, 2

Descriptive characteristics (initial $NH_4^+{}_{max}$) of the overall study sample stratified by scavenger treatment. In the overall sample, initial $NH_4^+{}_{max}$ was not associated with use of a specific scavenger treatment for the attenuated phenotype ($p=0.30$ for attenuated phenotype), however height of initial $NH_4^+{}_{max}$ was associated with use of a specific scavenger treatment for the severe phenotype ($p=0.014$). Disease-specific analyses revealed an association between higher initial $NH_4^+{}_{max}$ and use of a bi-scavenger treatment for severely affected individuals with CTLN1 ($p<0.01$), whereas no association between height of initial $NH_4^+{}_{max}$ and use of a specific scavenger treatment was observed for all other

investigated disease-specific attenuated or severe phenotypes (each $p>0.05$). Two-sample Fisher-Pitman Permutation Test was applied. ASA, argininosuccinic aciduria; CTLN1, citrullinemia type 1; initial $NH_4^+{}_{max}$, initial peak plasma ammonium concentration; mOTC-D, male ornithine transcarbamylase-deficiency; SD, standard deviation.

Suppl. Table 3. Disease-specific scavenger treatment and HAEs

HAEs per year of observation<br>Mean, SD<br>Median, Min, Max, n			
**Attenuated**			
	**Benzoate**	**Butyrate**	**Benzoate & Butyrate**
**mOTC-D**	0.18, 0.13<br>0.13, 0.07, 0.39, 5	0.73, 0.92<br>0.50, 0.03, 3.96, 24	0.42, 0.27<br>0.25, 0.20, 0.73, 5
**CTLN1**	0.18, 0.15<br>0.13, 0.08, 0.44, 5	0.94, 1,65<br>0.26, 0, 6.18, 15	0.35, n.a.<br>0.35, 0.35, 0.35, 1
**ASA**	n.a.	n.a.	n.a.
**Severe**			
	**Benzoate**	**Butyrate**	**Benzoate & Butyrate**
**mOTC-D**	n.a.	n.a.	n.a.
**CTLN1**	1.38, 0.63<br>1.73, 0.66, 1.76, 3	0.52, 0.61<br>0.34, 0.03, 2.49, 18	0.85, 0.55<br>0.83, 0.27, 1.59, 6
**ASA**	1.36, 1.34<br>0.83, 0.19, 3.28, 7	0.95, 1.54<br>0.32, 0.12, 6.51, 19	0.11, n.a.<br>0.11, 0.11, 0.11, 1

Disease-specific scavenger treatment and HAEs. In severely affected individuals with CTLN1, use of Butyrate (as monotherapy or bi-scavenger treatment) compared to Benzoate might be associated with a trend towards lower HAEs per year of observation (p=0.077). No further association between use of a specific scavenger or bi-scavenger therapy and HAEs per year of observation was found (p=0.33 for attenuated mOTC-D; p=0.31 for attenuated CTLN1; p=0.53 for severe ASA). Two-sample Fisher-Pitman Permutation Test was applied. ASA, argininosuccinic aciduria; CTLN1, citrullinemia type 1; HAEs per year of observation, hyperammonemic events per year of observation; mOTC-D, male ornithine transcarbamylase-deficiency; SD, standard deviation.

Suppl. Table 4. Disease-specific scavenger treatment and motor abnormality

**Motor abnormality** Yes / No			
**Attenuated**			
	**Benzoate**	**Butyrate**	**Benzoate & Butyrate**
**mOTC-D**	2 / 9	4 / 17	0 / 5
**CTLN1**	1 / 4	1 / 12	0 / 3
**ASA**	n.a.	n.a.	n.a.
**Severe**			
	**Benzoate**	**Butyrate**	**Benzoate & Butyrate**
**mOTC-D**	n.a.	n.a.	n.a.
**CTLN1**	3 / 3	6 / 11	2 / 3
**ASA**	0 / 6	7 / 12	0 / 2

Disease-specific scavenger treatment and motor abnormality at last regular follow-up. Independent from disease-severity, neither a specific long-term monotherapy (with Benzoate or Butyrate) nor a bi-scavenger therapy is associated with improved motoric outcome at last regular follow-up ($p=0.44$ for attenuated phenotype, $p=0.75$ for severe phenotype). Pearson's Chi-squared Test was applied. ASA, argininosuccinic aciduria; CTLN1, citrullinemia type 1; mOTC-D, male ornithine transcarbamylase-deficiency.

Suppl. Table 5. Disease-specific scavenger treatment and cognitive outcome

Cognitive outcome [cognitive standard deviation score (cSDS)] and Age at testing [years]<br>Mean, SD<br>Median, Min, Max, n				
**Attenuated**				
		**Benzoate**	**Butyrate**	**Benzoate & Butyrate**
**mOTC-D**	**cSDS**	0.32, 1.25<br>0.03, -1.00, 2.00, 6	-0.86, 1.67<br>-0.53, -4.00, 1.40, 19	-4.00, n.a.<br>-4.00, -4.00, -4.00, 1
	**Age at testing**	10.6, 6.4<br>9.5, 3.2, 22.3, 6	16.1, 7.1<br>17.9, 4.0, 30.0, 19	26.2, n.a.<br>26.2, 26.2, 26.2, 1
**CTLN1**	**cSDS**	-0.40, 1.27<br>-0.27, -2.07, 1.00, 4	-1.23, 1.27<br>-1.20, -3.80, 0.93, 13	-1.93, n.a.<br>-1.93, -1.93, -1.93, 1
	**Age at testing**	13.5, 11.1<br>11.7, 2.1, 28.4, 4	7.0, 7.4<br>3.6, 1.0, 29.3, 13	8.8, n.a.<br>8.8, 8.8, 8.8, 1
**ASA**	**cSDS**	n.a.	n.a.	n.a.
	**Age at testing**	n.a.	n.a.	n.a.
**Severe**				
		**Benzoate**	**Butyrate**	**Benzoate & Butyrate**
**mOTC-D**	**cSDS**	n.a.	n.a.	n.a.
	**Age at testing**	n.a.	n.a.	n.a.
**CTLN1**	**cSDS**	-3.19, 1.37<br>-3.07, -5.00, -1.47, 5	-2.13, 1.50<br>-2.07, -4.00, 0.53, 14	-3.13, 0.83<br>-3.27, -4.00, -2.00, 4
	**Age at testing**	14.7, 6.3<br>16.5, 8.0, 22.2, 5	15.2, 10.1<br>14.2, 0.5, 39.0, 14	12.2, 8.2<br>12.7, 2.2, 21.4, 4
**ASA**	**cSDS**	-1.64, 1.43<br>-1.70, -3.87, 0.33, 6	-2.18, 1.15<br>-2.60, -3.47, 0.33, 17	n.a.
	**Age at testing**	7.1, 9.1<br>3.3, 0.6, 24.5, 6	9.2, 8.0<br>8.4, 0.5, 24.2, 17	n.a.

Disease-specific scavenger treatment and cognitive outcome. Independent from disease-severity, use of a specific (monoscavenger or bi-scavenger) long-term treatment is not associated with an improved cognitive outcome in age-adjusted individuals with mOTC-D, CTLN1 or ASA, as reflected by cSDS ($p=0.12$ for attenuated mOTC-D, $p=0.26$ for attenuated CTLN1; $p=0.23$ for severe CTLN1, $p=0.35$ for severe ASA). Two-sample Fisher-Pitman Permutation Test was applied. ASA, argininosuccinic aciduria; CTLN1, citrullinemia type 1; cSDS, cognitive standard deviation score; HAEs per year of observation, hyperammonemic events per year of observation; mOTC-D, male ornithine transcarbamylase-deficiency; SD, standard deviation.

Suppl. Table 6. Scavenger treatment and growth

Growth [SDS]<br>Mean, SD<br>Median, Min, Max, n			
**Attenuated**			
	**Benzoate**	**Butyrate**	**Benzoate & Butyrate**
**Overall (mOTC-D and CTLN1)**	-0.17, 0.91<br>-0.30, -2.53, 3.06, 72	-0.27, 1.04<br>-0.23, -3.01, 2.89, 206	-0.49, 0.78<br>-0.48, -2.17, 1.32, 23
**Severe**			
	**Benzoate**	**Butyrate**	**Benzoate & Butyrate**
**Overall (CTLN1 and ASA)**	-1.61, 1.32<br>-1.66, -4.94, 2.03, 72	-1.19, 1.11<br>-1.10, -4.86, 1.41, 204	-0.92, 0.72<br>-0.73, -2.75, 0.17, 44

Scavenger treatment and growth development within individual observation periods. Overall 42 individuals with an attenuated disease course (mOTC-D and CTLN1) receiving Benzoate (n=11), Butyrate (n=28) or Benzoate & Butyrate (n=3) were evaluated comprising altogether 301 individual observation points. Moreover, overall 55 individuals with a severe disease course (CTLN1 and ASA) receiving Benzoate (n=14), Butyrate (n=33) or Benzoate & Butyrate (n=8) were evaluated comprising altogether 320 individual observation points. Descriptive characteristics are depicted above. Height development within the patients' individual observation periods are stratified by disease-severity and are depicted in each long-term scavenger treatment group separately (Benzoate vs. Butyrate vs. Benzoate & Butyrate; **Fig. 4**). ASA, argininosuccinic aciduria; CTLN1, citrullinemia type 1; mOTC-D, male ornithine transcarbamylase-deficiency, SD, standard deviation.

Suppl. Table 7. Additional members and affiliations of the Urea Cycle Disorders Consortium (UCDC) and the European registry and network for Intoxication type Metabolic Diseases (E-IMD)

**UCDC consortium (to be listed in PubMed in alphabetical order)**
Nicholas Ah Mew, Children's National Health System, The George Washington School of Medicine, Washington, DC, USA
Lindsay C. Burrage, Department of Molecular and Human Genetics, Baylor College of Medicine and Texas Children’s Hospital, Houston, Texas, USA
Gerard T. Berry, Harvard Medical School and Boston Children’s Hospital, Boston, Massachusetts, USA
Margo Breilyn, Department of Human Genetics and Genomic Sciences, Icahn School of Medicine at Mount Sinai, New York, NY, USA
Andreas Schulze, The Hospital for Sick Children and University of Toronto, Toronto, Ontario, Canada
Susan A. Berry, University of Minnesota, Minneapolis, Minnesota, USA
Derek Wong, David Geffen School of Medicine at UCLA, Los Angeles, California, USA
Matthias R. Baumgartner, Division of Metabolism and Children’s Research Center, University Children’s Hospital Zurich, University of Zurich, Switzerland
Laura Konczal, University Hospitals Cleveland Medical Center, Cleveland, Ohio, USA
Can Ficicioglu, Children’ Hospital of Philadelphia, Philadelphia, USA
Curtis R. Coughlin II, Associate Faculty of Bioethics, University of Colorado, Aurora, CO, USA
Gregory Enns, Stanford University Medical Center, Stanford, CA, USA
Renata C. Gallagher, Division of Medical Genetics, Department of Pediatrics, University of California, San Francisco, CA, USA
Cary O. Harding, Department of Molecular & Medical Genetics, Oregon Health & Science University, Portland, OR, USA
Christina Lam, Seattle Children’s Hospital, University of Washington, Seattle, Washington, USA
Nicola Longo, University of California Los Angeles, Los Angeles, CA, USA

Shawn E. McCandless, Children’s Hospital Colorado, University of Colorado School of Medicine, Aurora, CO, USA
Jennifer Seminara, Children's Research Institute, Children's National Health System, Washington, DC, USA
Tamar Stricker, Division of Metabolism and Children’s Research Center, University Children’s Hospital Zurich, University of Zurich, Switzerland
Greta Wilkening, Children’s Hospital Colorado, Aurora, CO, USA

E-IMD registry (to be listed in PubMed in alphabetical order)
Carlo Dionisi-Vici, Ospedale Pediatrico Bambino Gesù IRCCS, U.O.C. Malattie Metaboliche, Rome, Italy
Dries Dobbelaere, Centre de Référence Maladies Héréditaires du Métabolisme de l'Enfant et de l'Adulte, Jeanne de Flandre Hospital, CHRU Lille, and RADEME EA 7364, Faculty of Medicine, University Lille 2, Lille, France
Angeles Garcia-Cazorla, Hospital San Joan de Deu, Institut Pediàtric de Recerca. Servicio de Neurologia and CIBERER, ISCIII, Barcelona, Spain
Elaine Murphy, National Hospital for Neurology and Neurosurgery, London, United Kingdom
Tomáš Honzík, General Faculty Hospital in Prague, Prague, Czech Republic
Corinne De Laet, Hôpital Universitaire des Enfangs Reine Fabiola – Université Libre de Bruxelles, Bruxelles, Belgium
Elisa Leão Teles, Unidade de Doenças Metabólicas, Serviço de Pediatria, Centro Hospitalar Universitário de S. João EPE, Porto, Portugal
Allan M. Lund, Centre Inherited Metabolic Diseases, Departments of Paediatrics and Clinical Genetics, Copenhagen University Hospital, Rigshospitalet, Copenhagen, Denmark
Aline Cano, Reference Centre for Inborn Errors of Metabolism, La Timone Enfant University Hospital, APHM, Marseille, France
Ute Spiekerkoetter, Department of General Pediatrics, Adolescent Medicine and Neonatology, Medical Center - University of Freiburg, Faculty of Medicine, Freiburg, Germany
Yin-Hsiu Chien, National Taiwan University Hospital, Department of Medical Genetics and Pediatrics, Taipei, Taiwan
Adrijan Sarajlija, Mother and Child Health Care Institute of Serbia, Department of Metabolism and Clinical Genetics and University of Belgrade, School of Medicine, Belgrade, Serbia
Daniela Karall, Medical University of Innsbruck, Clinic for Pediatrics, Innsbruck, Austria
Martin Lindner, Department of Pediatrics, Division of Neuropaediatrics, Goethe University Frankfurt, Frankfurt (Main), Germany
Pascale de Lonlay, Centre de référence et service de maladies héréditaires du métabolisme, Hôpital Universitaire Necker, APHP, Filière G2M, MetabERN, Université de Paris, Paris, France
Consuelo Pedrón-Giner, Sección de Gastroenterología y Nutrición, Hospital Infantil Universitario Niño Jesús, Madrid, Spain
